# Bioprinting the Craniofacial Region: A New Era in Regenerative Medicine and Dentistry

**DOI:** 10.3390/jfb17090459

**Published:** 2026-09-08

**Authors:** Duaa Abuarqoub, Mais Emad, Alqassem H. Abuarqoub, Majd F. Ellauzi, Tala Maragha

**Affiliations:** 1 Faculty of Pharmacy and Medical Sciences, University of Petra, Amman 11196, Jordan; mais999@hotmail.co.uk; 2 Department of Biotechnology, Faculty of Allied Medical Sciences, Al-Ahliyya Amman University, Amman 19111, Jordan; aha87@georgetown.edu; 3 Al Wafi Academy, Amman 11190, Jordan; majd.ellauzi@me.com; 4 Faculty of Dentistry, University of Petra, Amman 11196, Jordan; tala.maragha@uop.edu.jo

**Keywords:** 3D bioprinting, craniofacial, regenerative medicine

## Abstract

Background: Three-dimensional (3D) printing and bioprinting have emerged as transformative technologies in tissue engineering and regenerative medicine. Conventional 3D printing enables the fabrication of complex, customizable scaffolds that mimic the native extracellular matrix (ECM), providing mechanical support essential for cell adhesion, proliferation, and differentiation. In contrast, 3D bioprinting integrates biomaterials, growth factors, and living cells into bioinks, allowing the precise layer-by-layer construction of tissue-like structures. Objective: This review aims to highlight recent advances and applications of both conventional 3D printing and 3D bioprinting in regenerative medicine, with a specific focus on craniofacial tissue regeneration. Particular emphasis is placed on the distinct roles of scaffold-based and cell-laden approaches. Methods: A comprehensive analysis of the current literature was conducted to examine both conventional 3D printing and bioprinting approaches used in craniofacial tissue engineering. Emphasis was placed on printing techniques, bioink composition and selection, scaffold design, and cell sources, as well as their applications across multiple tissue types. Results: Recent developments demonstrate that conventional 3D printing enables the fabrication of customizable scaffolds, whereas 3D bioprinting enables the generation of highly organized, cell-laden constructs that closely resemble native tissue architecture. These technologies have shown promising outcomes in developing patient-specific disease models and regenerative implants for craniofacial tissues, including bone, dental, tracheal, ocular, muscle, and neural tissues. Conclusions: 3D printing and bioprinting offer powerful platforms for craniofacial regenerative medicine. Ongoing advancements in bioink formulation, printing precision, and biological integration are expected to further enhance their translational and clinical potential.

## 1. 3D Printing and 3D Bioprinting

Three-dimensional (3D) printing has significantly advanced tissue engineering by enabling the production of hydrogel-based scaffolds that closely resemble the natural ECM and provide a favorable environment for cell growth and differentiation [1]. 3D-printed scaffolds are used in regenerative medicine as both cell-free scaffolds and post-seeded scaffolds. These post-seeded scaffolds are conventionally printed scaffolds onto which cells are subsequently seeded after fabrication. Recent innovations have improved the functionality of these scaffolds for various applications, including wound healing, bone and cartilage regeneration. This regenerative potential was driven by the introduction of bioinks containing living cells, growth factors, and nanoparticles in a process referred to as bioprinting [2,3,4,5].

Bio-fabrication, an emerging field that has recently been gathering attention across various disciplines, involves the generation of biologically functional products in an automated manner. The process of bio-fabrication typically uses bioactive molecules, living cells, cell aggregates such as microtissues, or hybrid cell material constructs. Following the seeding process, these materials then undergo a maturation process to convert them into tissues [2]. More recently, bioprinting has been identified as a novel technique that can significantly improve the architectural control of the resulting biofabricated structures in a highly reproducible and standardized manner [3,4,5].

**Figure 1:**
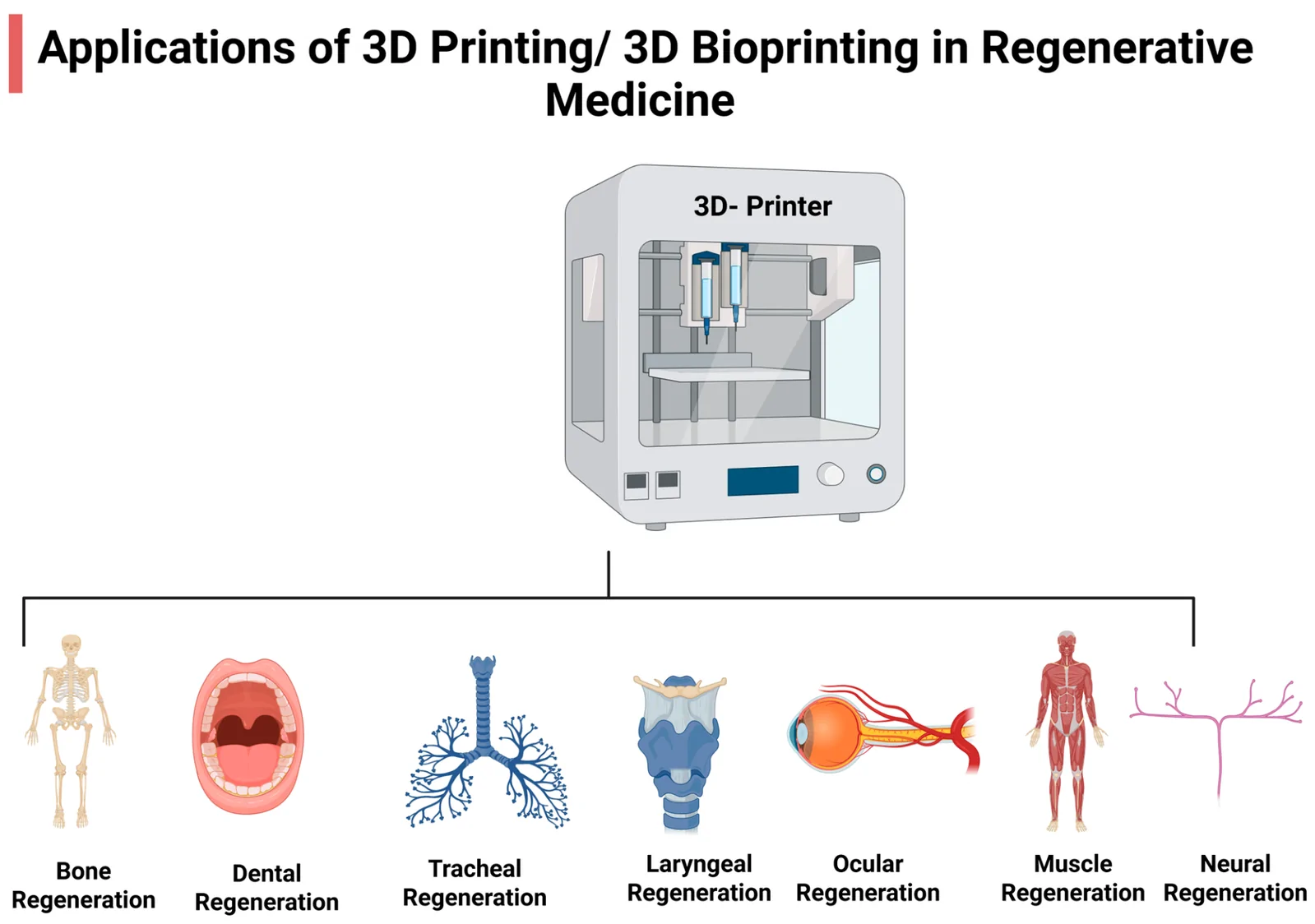
Applications of 3D Printing/3D Bioprinting in Regenerative Medicine. 3D printing and 3D bioprinting were successfully used to print different types of tissues, including bone tissue, dental tissue, tracheal tissue, laryngeal tissue, ocular tissue, muscle tissue, and neural tissue.

In its essence, 3D bioprinting facilitates the fabrication process of complex structures with spatial heterogeneities that closely resemble the natural structure of the tissue being replicated [6]. In comparison to traditional tissue engineering techniques that often involve solvent casting, freeze drying, gas foaming, phase separation, and electrohydrodynamic methods [6,7], bioprinting was found to demonstrate superior performance in terms of spatially controlling the nano-, micro-, and macrostructures of cell-laden constructs. Such superior performance has been attributed to the ability of 3D-bioprinted structures to synchronize the deposition and crosslinking of bioink with the motorized stage movement. This precise and reliable process results in 3D tissue constructions with predictable patterns and layouts comprising biomaterials (referred to as bioink) and/or living cells [8], as shown in Figure 1.

Despite the numerous advantages and strengths of tissue bioprinting, several challenges and gaps in available knowledge and evidence remain unaddressed, thereby hindering the scaling potential of bioprinting technology. Among these challenges are the complexity of target tissues, post-print tissue maintenance and maturation, and the lack of vasculature, nervous regulation, lymphatic drainage, and multiple supporting cell types necessary for cell growth and differentiation [9]. Further, the lack of strategic planning for uniform and scalable manufacturing of 3D-bioprinted structures, and the absence of a defined regulatory framework for bioprinted constructs were identified as barriers to increased uptake of 3D-bioprinting technology [9]. Furthermore, access to bioprinting technology may be severely limited by the rising cost of commercial 3D bioprinters and bioinks [10]. The high cost of bioinks is reflective of the complexity involved in the processes of synthesizing bioinks that ensure printability, shape fidelity, and structural integrity, while simultaneously maintaining high cell viability, proliferation, and tissue maturation [11]. Therefore, the need to continuously refine bioprinting methods, create novel biomaterials, and incorporate microvascular networks becomes evident to facilitate knowledge translation into clinical applications in tissue engineering.

Despite being in its early phases of research, the versatility of bioprinting has accelerated tissue engineering applications [8]. Available evidence categorizes these applications by the bioprinting method into three categories: (1) extrusion-based bioprinting, (2) inkjet/droplet bioprinting, and (3) laser-assisted bioprinting (LaBP) [12]. The choice of the bioprinting method is one of the factors that determine the selection of the appropriate bioink, in addition to considerations around the type of cells and the specific application and target tissue of the bioprinting process [13]. While the parameters and the workflow involved in each bioprinting method are unique, all techniques use computer-aided design/computer-aided manufacturing (CAD/CAM) systems to program 3D structures. Further, all techniques involve crosslinking or stabilization of bioinks during or after bioprinting to create the final morphologies of the intended tissue constructs [13].

In summary, 3D printing methods are widely defined based on whether living cells, growth factors, or hormones are integrated into the printing material during the printing process. Conventional 3D printing results in biological component-free scaffolds or constructs that lack biological materials in the printing ink. A scaffold can be post-seeded with cells after it has been created. In contrast, 3D bioprinting involves the deposition of a biological material-containing bioink, in which, for example, living cells are incorporated into the printing substance throughout the manufacturing process. This distinction is used consistently throughout the review to distinguish 3D printed, or 3D bioprinted, materials.

## 2. Bioink Selection

**Figure 2:**
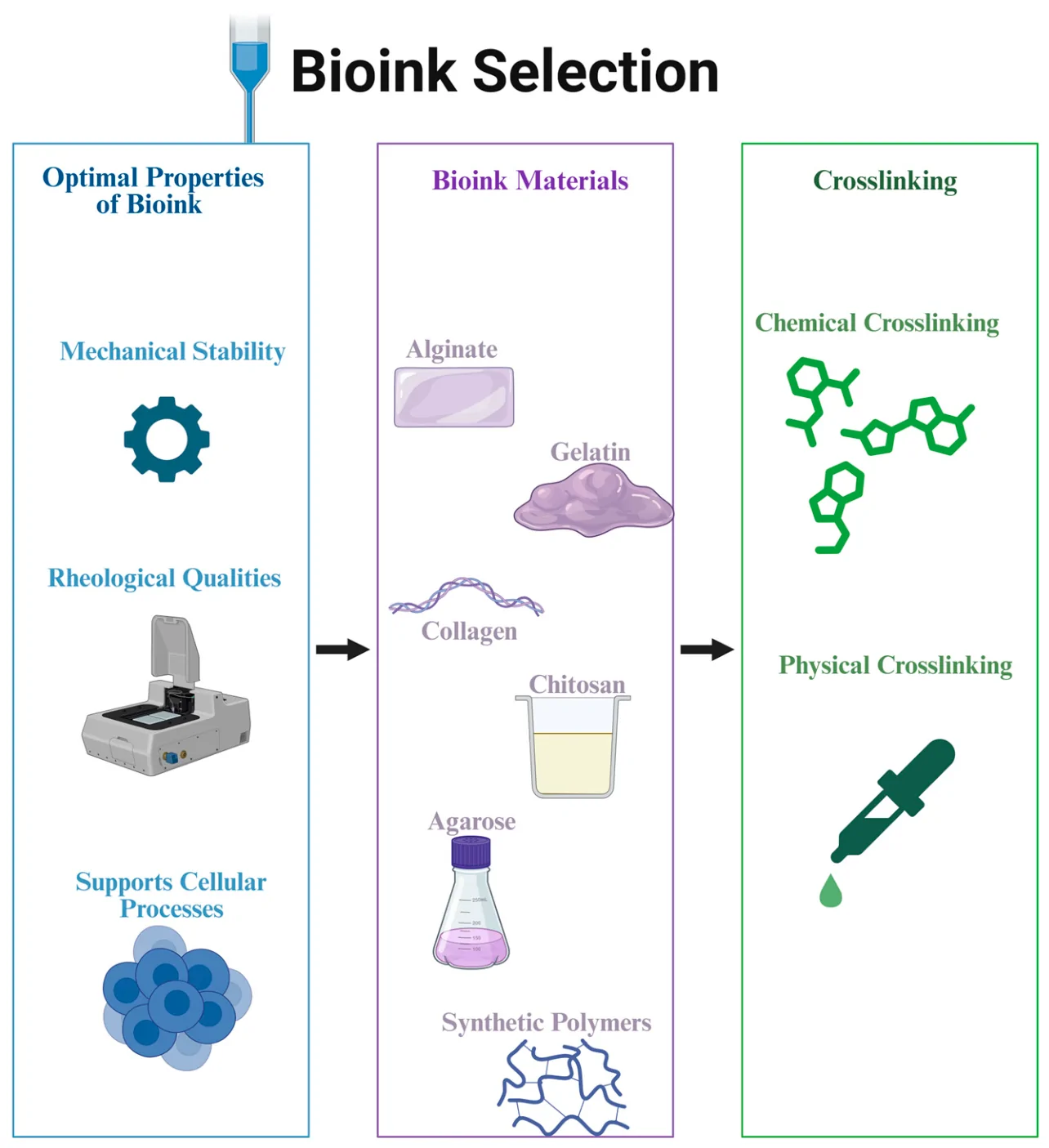
Bioink Selection. The main parameters of choosing a bioink, including the properties of an optimal bioink, the materials of a bioink, and types of crosslinkers, are represented in this figure.

A key step to ensure a successful bioprinting outcome is choosing the biomaterials for the bioink, which should be carried out early on in the planning phase of bioprinting processes. This step is necessary to guarantee printability and long-term usability of the resulting structures, as bioinks are typically designed to demonstrate biocompatibility and physical properties specific to the intended application [14,15,16]. The ideal bioink, as shown in Figure 2, is described as having the necessary mechanical, rheological, chemical, and biological qualities for optimal balance of printability and biological support [12]. Pluronic, polyethylene glycol (PEG), gelatin, and other shear-thinning biomaterials are examples of materials that are frequently used in the composition of bioinks. While these materials exhibit liquid-like behavior under high shear stress during the extrusion process, they would rapidly regain their gel state once bioprinted, preventing the structure from collapsing [17,18,19].

To date, it has been shown that hydrogels synthesized from natural biopolymers, such as alginate, gelatin, collagen, fibrin, hyaluronic acid, chitosan, and agarose, as well as numerous synthetic polymers, such as (PEG) and Pluronic, fulfill certain essential requirements as bioink materials, including optimal rheology to ensure printability, shape fidelity, and structural integrity. Simultaneously, these materials were found to preserve high cell viability, proliferation, and tissue maturation [4,11], which are important features for functional tissues that will only develop when cells survive the printing process and continue to proliferate within the construct. These factors have a direct impact on the tissue’s long-term viability, biological activity, and structural integrity [20].

The significance of selecting bioinks goes well beyond the preliminary stages of bioprinting, as it has been found to determine the long-term stability of the bioprinted tissue constructs. Specifically, the type of bioink often determines the secondary crosslinking mechanism that will be utilized to further stabilize the bioprinted structures [20]. Crosslinking occurs through physical or chemical mechanisms, with physical crosslinking being associated with non-covalent interactions such as thermally induced sol–gel transitions or ionic contacts. Conversely, chemical crosslinking is associated with the production of new covalent bonds [21]. Physically crosslinked gels are often subject to dissolution and are typically unstable over extended periods of time. Chemically crosslinked gels, on the other hand, are described as being more stable over the long term and can serve as the biomimetic extracellular matrix in constructive bioprinting [4].

Among the early attempts to develop bioinks and bioprint structures was described in Kolesky et al.’s study [22], which introduced a novel approach for employing 3D bioprinting to create complex structures. To replicate the complexity of natural tissues, tissue constructs used in the study’s methods combined several cell types, extracellular matrix elements, and vascular networks. These components were then organized in consecutive layers using a co-axial extrusion-based bioprinting technique. Cell types included in the study’s bioink were human mesenchymal stem cells (hMSCs), human umbilical vein endothelial cells (HUVECs), and human neonatal dermal fibroblasts (hNDFs). In addition to cellular components, gelatin, alginate, and fibrinogen were also incorporated in the study’s bioink. The study’s results were considered a proof of concept for bioprinting tissue constructs with diverse cell populations and distinct circulatory networks. These structures showed promising properties for application in tissue engineering, regenerative medicine, and drug screening. In summary, Kolesky et al.’s study [22] presented a method for producing complex tissue constructs using distinct cell types and extracellular matrix components. The ability to incorporate vascular networks in the bioprinted structures addresses one of the major challenges in bioprinting, as it ensures that the resulting constructs are not only structurally integral, but also functional [22]. This development was reflected in the wide range of bioprinting applications in regenerative medicine and dentistry.

## 3. Applications of 3D Bioprinting in the Craniofacial Region

### 3.1. Bone and Cartilage Regeneration

**Figure 3:**
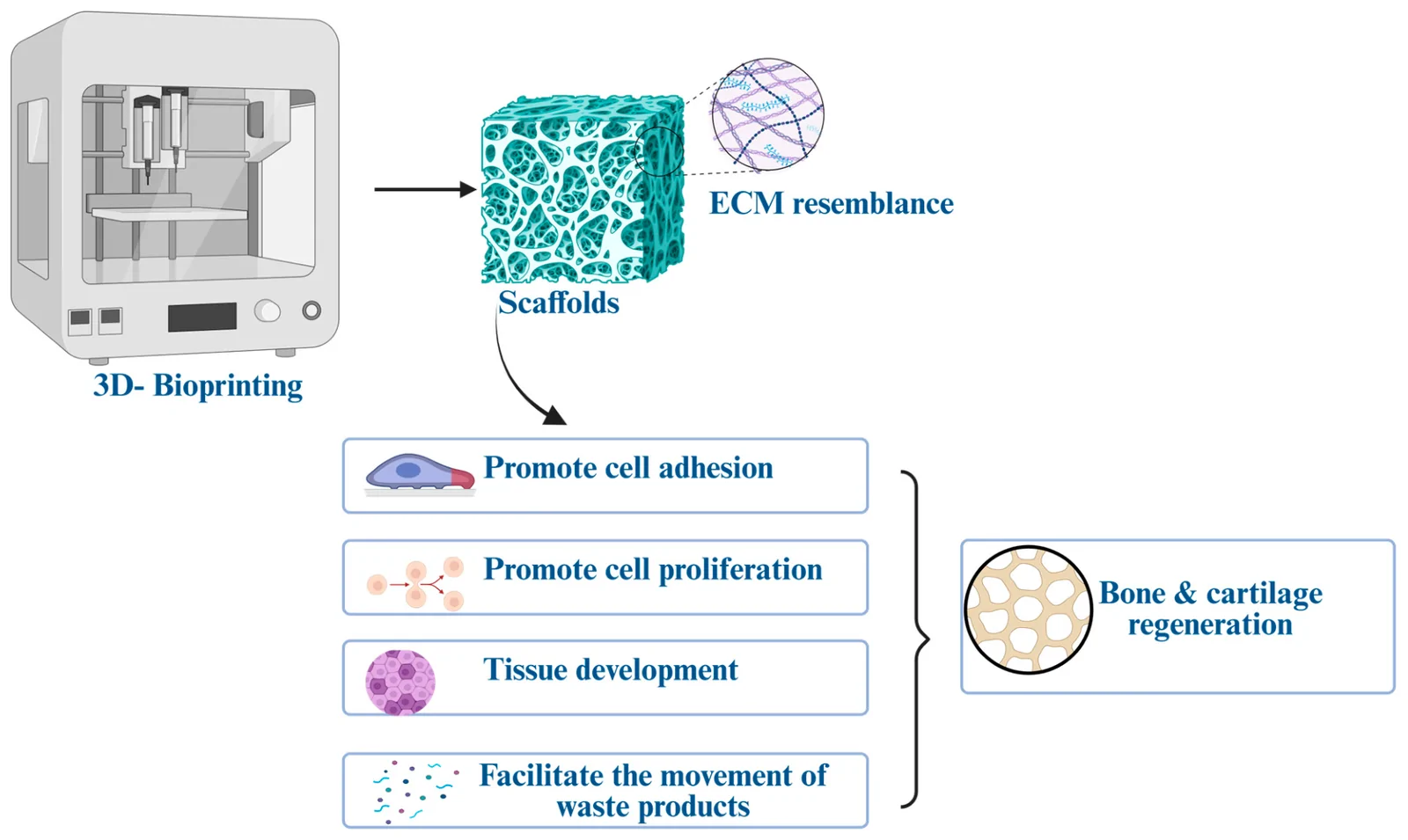
The 3D-Bioprinted ECM Resembling Scaffolds. Three-dimensional scaffolds are made to resemble the Extracellular matrix (ECM) of natural tissues in order to promote cell adhesion, proliferation, tissue development, and facilitate the movement of waste products. Consequently, these scaffolds regenerate defective bone and cartilage.

Three-dimensional scaffolds, as shown in Figure 3, are made to resemble the ECM of natural tissues in order to promote cell adhesion, proliferation, and tissue development [23]. These scaffolds facilitate the movement of waste products and nutrients while guiding tissue regeneration and offering mechanical support [24].

In 2024, Yu et al. [25] developed a 3D bioprinted scaffold that employs stem cells and cell-derived vesicles to produce a functional bioink that treats periodontitis-induced bone abnormalities. Given their strong immunomodulatory and regenerative properties, human periodontal ligament stem cells (hPDLSCs) were selected as the cellular component of the bioink utilized in this study. The bioink was formulated using a gelatin-alginate-silk fibroin (GAS) composite that demonstrated optimal printability, biocompatibility, and mechanical stability. Using an extrusion-based 3D bioprinting method, the scaffold was fabricated, enabling the cell-rich bioink to be precisely deposited layer by layer. Following bioprinting, a dual crosslinking process was initiated to reinforce the mechanical and structural integrity of the scaffold: Ionic crosslinking with calcium chloride (CaCl_2_) for the alginate network, and enzymatic crosslinking using horseradish peroxidase (HRP) with hydrogen peroxide (H_2_O_2_) for the gelatin and silk fibroin phase.

The in vitro component of this study [25] included osteogenic differentiation analysis (ALP and alizarin red staining, qRT-PCR for osteogenic gene markers), cell viability and proliferation testing (Live/Dead staining, CCK-8 assay), and endothelial cell tube formation assays for angiogenic potential evaluation. The in vivo component of the same study, on the other hand, involved the implantation of the 3D-bioprinted GAS-hPDLSC scaffold into a rat model with a periodontitis-induced bone defect. Histological staining (H&E, Masson’s trichrome), immunohistochemistry for osteogenic and angiogenic markers, and micro-CT imaging were used to assess bone regeneration. The results indicate that the bioprinted scaffold significantly enhanced osteogenesis, angiogenesis, and tissue integration, suggesting a promising bioprinting method for the regeneration of alveolar bone in the maxilla and mandible [25].

In addition to investigating the regenerative potential of bioprinted structures in conditions resembling healthy patients, Wang et al.’s study [26] explored the potential of healing bone defects in the microenvironment of diabetes, typically characterized by high levels of oxidative stress and hyperglycemia. A novel TZGP (α-TCP/ZnO/GM@P2) composite scaffold was designed with the intended application of bone regeneration under diabetic environments. The scaffold integrated ZnO nanoparticles for antibacterial and antioxidant properties with α-tricalcium phosphate (α-TCP) cement for structural support. Gelatin microspheres that encapsulate and shield P2, a new parathyroid hormone-related peptide, were also incorporated into the bioink. Together, these components provided complementary benefits, where the scaffold demonstrated high levels of bioactivity and biocompatibility, and the mechanical properties of cancellous bone, including its strength, were preserved. Following the creation of the bioink, a 3D printing-assisted extrusion approach was used to fabricate the TZGP scaffold, which allowed an accurate layer-by-layer deposition of α-TCP paste containing P2-loaded gelatin microspheres and ZnO nanoparticles. Given the accurate control over pore size, porosity, and structural interconnectivity provided by this 3D printing method, bioactive components were distributed uniformly, and Zn^2+^ ions and P2 peptides were released continuously. The experimental findings from both in vitro and in vivo investigations revealed that the 3D-printed TZGP scaffold reduced reactive oxygen species accumulation, mitigated DNA damage, and preserved mitochondrial stability. Further, the scaffold was able to promote cell proliferation, migration, and osteogenic-angiogenic differentiation under high glucose conditions typically present in diabetic patients. The TZGP construct is a promising 3D-printed biomaterial that can be utilized for effective repair of diabetic bone defects in the head and neck areas and beyond, since it demonstrated greater biodegradability, faster tissue integration, and enhanced bone formation and vascularization when compared to traditional α-TCP scaffolds [26].

Glass-based biomaterials were also utilized in bioprinting. In a recent study by Li et al. [27], excellent bone growth was observed in a rabbit model utilizing 3D-printed scaffolds made of the ICIE16 glass formulation (49.46 mol% SiO_2_, 36.6 mol% CaO, 6.6 mol% Na_2_O, 6.6 mol% K_2_O, and 1.07 mol% P_2_O_5_). This particular composition could be extruded into scaffolds without crystallizing, given the amorphous structure of bioactive glass. Generally, it is recommended to limit crystallization during the extrusion of bioinks, as it could make the material brittle, difficult to process, and less bioactive, ultimately limiting the scaffold’s potential to support bone regeneration [28]. Therefore, the absence of crystallization in glass-based bioinks was found to maintain their biological performance, mechanical integrity, and printability [28].

Following the extrusion of the glass-based and cell-free bioink in 3D structures, human bone marrow stem cells (hBMSCs) were cultured onto the 3D-printed ICIE16 scaffolds to explore their potential in promoting cell proliferation and osteogenic differentiation in vitro, both with and without osteogenic supplements. The results were compared to a control group in which cells were cultured in media with only dissolution products of bioactive glass. The hypothesis was that despite the absence of osteogenic supplements, direct cell culture on the scaffolds would promote osteogenic differentiation more rapidly than exposure to dissolution products alone. Indeed, when compared to cells in monolayer culture with glass dissolution media over a six-week period, hBMSCs cultured on ICIE16 scaffolds were found to show significantly higher expression of osteogenic and extracellular matrix markers, including Runx2, Col1a1, Osteopontin, Osteocalcin, and Alkaline Phosphatase, in comparison to the control group. Early differentiation (weeks 2–4) was further enhanced by the addition of osteogenic supplements. However, by week 6, the levels of marker expression in the supplemented and non-supplemented groups were comparable. These results confirm that both the 3D architecture and ionic dissolution of ICIE16 bioactive glass play distinct and synergistic roles in promoting osteogenic differentiation of hBMSCs [27].

In addition to bone-generating scaffolds, an emerging area in 3D printing for bone-related applications is bony implants. A study conducted by Dahl, Jensen, Poujade, and associates aimed to create resorbable 3D-printed bone implants that can be used in both in vitro and in vivo settings. The researchers created scaffolds with tailored macro- and micro-porosity to resemble natural bone structure using Ossi Ink, a bio-ink based on β-tricalcium phosphate (β-TCP). Structures were printed using an extrusion-based method and then sintered to solidify the material and provide mechanical stability. Osteoblasts and other bone-forming cells were seeded onto the scaffolds for in vitro investigations to evaluate biocompatibility, viability, and osteogenic activity. In vivo, the printed structures were implanted into surgically generated bone deficiencies, which were mostly carried out in pig mandible models. Imaging and histological examinations over an interval of several months demonstrated progressive degradation of the β-TCP scaffold along with vascularization, tissue remodeling, and new bone formation, suggesting successful integration into host tissue. These implants’ potential in driving customized bone regeneration is illustrated by their printability, biodegradability, and structural control, which connects laboratory research with preclinical regenerative applications [29].

Several bioprinting advancements were identified for other applications in the skeletal system, including cartilaginous regeneration, with some dating back to the early 2010s. One prominent example is Markstedt et al.’s study, where human chondrocytes and a new nanocellulose-alginate (NFC–alginate) bioink were utilized to bioprint cartilage-like structures. The bioink was composed of sodium alginate, a naturally occurring polysaccharide that promotes rapid ionic crosslinking and mechanical stability, and nanofibrillated cellulose (NFC), which provides high viscosity and excellent shear-thinning qualities for smooth extrusion. Following printing, CaCl_2_ was used to crosslink the constructs, where the ionic gelation of the alginate component was induced, thereby forming a stable and porous three-dimensional network that could facilitate nutrient diffusion and cell viability. The resulting imaging data demonstrate the deposition of cell-rich NFC-alginate bioink onto predesigned shapes, including grid structures, cylindrical scaffolds, and anatomically accurate models like a sheep meniscus and a human ear. Cell viability and morphology were evaluated by live/dead staining and confocal microscopy after printing. The results showed that over 73% of chondrocytes were still viable after printing, and more than 85% were still viable after 7 days of culture, suggesting that shear-induced cell damage was negligible. Histological and microscopic examinations verified that the encapsulated chondrocytes retained the rounded shape of healthy cartilage cells, and the printed constructs showed minimal cytotoxicity and retained their shape fidelity. Thus, the study revealed that the NFC-alginate bioink crosslinked with CaCl_2_ provides the optimal possible balance between printability, biocompatibility, and mechanical stability, making it a viable platform for cartilage tissue 3D bioprinting [30].

In 2019, Prasopthum et al. [31] introduced a quick and single-step 3D printing method that uses direct ink writing of an agitated viscous polymer solution to create polymer scaffolds with microstruts (~60 μm) that have micro- and nanoscale surface pores (0.2–2.4 μm). The pore size, density, and alignment were accurately regulated by adjusting the printing speed or the level of agitation. These micro-/nanoporous scaffolds were found to improve the chondrogenic and osteogenic differentiation of mesenchymal stem cells (MSCs) even when soluble differentiation agents were not present. The study’s results also indicate that the structures’ topography also affected MSC adhesion, shape, and differentiation by lineage, depending on the differentiation medium. These results indicate that this technique provides an adaptable strategy for creating polymer scaffolds with customized architecture and surface characteristics that can be applied across various tissue engineering and regenerative medicine fields, including bone regeneration [31].

An important consideration in the area of cartilaginous regeneration is the complex and gradient-like structure of osteochondral tissue. These tissues exhibit noticeable differences in biological, physiological, and mechanical properties starting from the subchondral bone layer beneath the joint surface and ending with the hyaline cartilage layer at the surface. This structural complexity poses major challenges for developing effective tissue-engineered solutions for osteochondral defects. A study by Nowicki et al. utilized 3D printing technology to regenerate cartilage tissues, advancing bioinks as a possible approach to overcome these challenges [32]. The study intended to develop novel osteochondral tissue constructs by combining innovative bioink formulations with fused deposition modeling (FDM) 3D printing and casting processes. The osteochondral matrix was constructed using a shape-memory polycaprolactone (PCL)-based substance made of polycaprolactone-triol, castor oil, and poly-hexamethylene diisocyanate. Human mesenchymal stem cells (hMSCs) were cultured onto the printed structure, and chondrogenic growth factors were embedded in the cartilage layer. Nanocrystalline hydroxyapatite (nHA) was also produced and added to reinforce the subchondral bone layer. The study’s results illustrate that 3D printed structures with nHA and bioactive compounds demonstrated superior mechanical strength and encouraged better adhesion, proliferation, and differentiation of hMSCs. One major conclusion of this study is that region-specific chondrogenic regeneration can be achieved via customized bioink compositions coupled with an integrated casting process to efficiently regulate the mechanical properties and cellular responses of osteochondral structures [32].

ECM-based bioinks were also proposed as a component of bioinks used to regenerate human cartilage, given their ability to mimic the natural composition of human cartilage [33]. GelMA, hyaluronic acid methacrylate, and chondroitin sulfate methacrylate were among the modified proteins and polysaccharides that made up the bioink composite. Different assays were conducted to assess the functionality of the 3D bioprinted structure, including RNA sequencing (RNA-seq), examinations of morphology, swelling behavior, degradation profile, mechanical and rheological properties, printability, biocompatibility, and chondrogenic differentiation. The results demonstrated that the photocrosslinked ECM hydrogels had good printability, robust mechanical integrity, and acceptable degradation rates. Furthermore, the structural, biochemical, and chondrogenic properties of synovium-derived mesenchymal stem cells (SMSCs) were markedly enhanced by the 3D bioprinted ECM scaffolds, which also showed excellent form fidelity. Increased chondrogenic ECM deposition was found to be facilitated by the addition of transforming growth factor-beta 1 (TGF-β1), which further encouraged SMSCs aggregation, proliferation, and differentiation. The study’s results indicate that the combination of 3D bioprinted ECM scaffolds and TGF-β1 successfully supported cartilage repair and joint functional recovery, as demonstrated by the results of in vivo experiments and gait evaluation. In general, the photo-crosslinked ECM bioink presents a viable method for both the regeneration of injured cartilage and the 3D bioprinting of functioning cartilage tissue [33].

Moreover, bioprinting was proposed as an innovative therapeutic approach to restore not only the cartilaginous structure, but also the articular cartilage functions. In their 2019 study, Rubí-Sans et al. [34] combined a nanofibrous self-assembling peptide hydrogel (RAD16-I) with a robust mechanical PCL structure to construct a hybrid 3D scaffold that replicates the biological and structural complexity of native cartilage. By utilizing a 30% PCL solution to form porous grid structures with pore sizes of around 300 µm, the PCL scaffold was created via FDM 3D printing. The RAD16-I hydrogel was then incorporated into these structures to create a composite material. hBMSCs were seeded on these 3D structures and were later cultured under the conditions necessary for chondrogenic differentiation and growth. The structures and their functions were characterized through various methods, including scanning electron microscopy (SEM) for morphology characterization, surface hydrophilicity using water contact angle measurements, cytotoxicity and metabolic activity using MTT assays, viscoelastic properties using mechanical and rheological testing, and cartilage-related gene expression analysis using qRT-PCR. Collagen type II and aggrecan-rich extracellular matrix deposition were also verified by immunostaining. In comparison to PCL alone, the PCL/RAD16-I composite scaffolds showed superior mechanical properties, increased cell viability, and improved chondrogenic differentiation. Overall, this integrated bioengineered approach demonstrated great promise for articular cartilage regeneration by effectively combining bioactive microenvironments with the necessary structural support [34].

In examining the results of different studies exploring the role of stem cells in bioprinting, MSCs, including PDLSCs and BMScs, were found to promote bone and cartilage regeneration when seeded onto 3D bioprinted/3D printed constructs or directly incorporated into bioink. MSCs combined with hydrogel-based bioinks enriched with ECM components such as GelMA, hyaluronic acid methacrylate, and chondroitin sulfate methacrylate using FDM and extrusion-based bioprinting consistently displayed good structural fidelity, maintained cell viability, and promoted cellular differentiation and tissue development. In both bony and cartilaginous structures, 3D bioprinting/3D printing often demonstrated the most promising outcomes for functional regeneration. Overall, the findings summarized in Table 1 show that 3D printing and bioprinting can be modified to meet the unique structural and biological needs of bone, cartilage, and osteochondral regeneration. However, the techniques differ significantly in terms of biological complexity and translational maturity. For example, incorporating stem cells and cellular vesicles into printed bioinks was found to promote alveolar bone repair in periodontitis-induced defects [25]. Other materials, such as multi-component scaffolds designed to promote osteogenesis and vascularization, were found to be especially advantageous for bone regeneration under diabetic conditions [26]. Similarly, control over scaffold micro- and nanotopography has been shown to influence MSC differentiation [31], demonstrating that therapeutic results are determined by the design and topography of the construct, and not only its biological composition.

Given the wide range of materials, designs, and cell sources used for bone and cartilage regeneration purposes, caution should be exercised when comparing the results of these studies. Further, while the current literature seems to be focused largely on in vitro cellular responses or tissue development outcomes, there is a paucity of evidence around long-term functional regeneration using bioprinted structures, integration with native tissue, vascularization, and performance in clinically relevant animal models. These limitations are particularly relevant for complex or multi-component structures, where increasing biological functionality may be coupled with increased manufacturing complexity as well as issues with repeatability and uniformity. As a result, there is a need to validate such approaches via standardized and clinically relevant settings prior to their large-scale deployment as regenerative medicine options.

### 3.2. Dental Regeneration

Tooth loss or damage resulting from periodontal disease and dental caries poses a global burden on healthcare systems and can be detrimental to the quality of patients’ lives. While current treatments have enabled tooth restoration to a certain extent, achieving functional tooth regeneration remains a challenging task due to the intricate and hierarchically organized architecture and complex anatomical arrangement of teeth. Similar to bone tissue, the composition of dental tissues includes an ECM, more specifically, a dentin matrix, that has been shown to be associated with host signaling molecules that can promote odontogenesis. Various studies attempted to 3D print/bioprint different scaffolds with various types of stem cells to be applied as a regenerative tool in dentistry [35,36].

To fabricate a patient-specific dentin-pulp construct, researchers developed a hybrid 3D bioprinting technique that combines a thermoplastic scaffold (PCL) and a fibrin-based bioink loaded with human dental pulp stem cells (hDPSCs). Fibrinogen (5–20 mg/mL), gelatin, hyaluronic acid, and glycerol were included in the bioink’s composition. To examine the role of fibrinogen on the mechanical properties, printability, and cell viability (>88%) of the bioprinted structures, various fibrinogen concentrations were utilized [37]. Alizarin red staining and gene expression of the odontogenic markers DSPP and DMP-1 were used to measure cytocompatibility, proliferation, and differentiation. When examining the outcomes of this method, stronger odontogenic differentiation was found to be induced by higher concentrations of fibrinogen (stiffer bioink). Lower fibrinogen concentrations, on the other hand, were found to maintain larger numbers of undifferentiated cells. The study’s method included co-printing of PCL alongside bioinks with two different fibrinogen concentrations (high- and low-fibrinogen) in specific spatial arrangements using a custom bioprinter to maintain structural integrity. The PCL and bioink were then crosslinked with thrombin and cultured in differentiation media. After 15 days, localized mineralization in the outer dentin region and preserved undifferentiated pulp-like cells were observed towards the center, demonstrating spatial control of differentiation in a geometrically accurate and patient-specific tooth-like tissue [37].

In an attempt to closely resemble the natural environment of tooth tissues, sodium alginate was combined with both the soluble and insoluble parts of dentin matrix to develop a unique bioink. According to Athirasala et al. [35], a 1:1 mixture of alginate-dentin is suggested to provide the optimal balance of printability and biological support. When encasing odontoblast-like cells, this hybrid bioink maintained extremely high cell viability and enabled stable 3D extrusion bioprinting. Crucially, the results show that despite the absence of additional growth factors, soluble dentin-derived molecules (particularly at 100 µg/mL) remained well-retained in the bioprinted gel and significantly stimulated odontogenic differentiation of stem cells from the apical papilla [35].

In addition to exploring the potential of bioprinters in regenerating tooth structure, another area where 3D bioprinting has been rapidly advancing is the regeneration and restoration of tooth-surrounding support structure, also known as the periodontal ligament (PDL). A study conducted in 2023 [38] developed a bioactive composite scaffold for periodontal regeneration using extrusion-based 3D bioprinting. The study describes the development of a multi-component bioink consisting of bioactive glass microspheres (BGM), sodium alginate (SA), and GelMA. The BGM-loaded scaffolds demonstrated higher strength, slower disintegration, and in vitro mineralization (apatite production) compared to scaffolds without BGM. Initially, cell-free scaffolds were printed to evaluate their structure, swelling behavior, mechanical properties, and bioactivity. Next, mouse bone marrow with mesenchymal stem cells (mBMSCs) was mixed with growth factors (BMP-2 for bone and PDGF for soft tissue) in the bioink, bioprinted, and crosslinked with calcium (for alginate bioink) and UV (for GelMA bioink). The resulting structure was then evaluated to examine the viability, proliferation, and differentiation of the cellular component. The BMP-2/PDGF-loaded structures markedly increased the expression of osteogenic markers (OPN, OCN, Runx2) and fibronectin (a marker for soft tissue healing), and the cells demonstrated good viability rates after printing. Following the implantation of these scaffolds in beagle dogs with periodontal abnormalities, a strong regeneration of gingiva, periodontal ligament, and alveolar bone was observed at the eight-week mark, particularly in the group that received the bioink that contained both cells and growth factors [38].

Similarly, a study by Raveendran et al. [36] aimed to create and optimize a 3D bioprinting procedure for PDL cells. The methodology included examining various amounts of GelMA as the bioink basis and systematically testing different concentrations of this hydrogel precursor. PDL cell survival, print fidelity, and the structural stability of the bioprinted constructs were evaluated. The study’s results identified an ideal window of conditions and printing parameters that combined good cell viability with dimensional accuracy and high-resolution printing. These parameters included photoinitiator concentration (0.05% LAP), UV-exposure period (20 s), extrusion pressure (135 kPa), and needle diameter (25 G). The study’s results indicate that PDL cells can be consistently 3D bioprinted in a functional, stable scaffold under such conditions [36].

Furthermore, another study by Lin et al. [39] aimed to create a 3D-printed collagen-based microfibrous scaffold to facilitate PDL cell regeneration and resemble the PDL’s natural structure. The researchers created waveform (crimped) microfibers mimicking those found in natural PDL, in addition to straight microfibers from type I collagen using extrusion-based 3D bioprinting. When compared to straight fibers, PDL cells seeded on waveform fibers demonstrated improved viability, spreading, and adherence under shear stress. Cyclin D, E-cadherin, and periostin were found to be upregulated by gene expression analysis and were identified as indicators associated with cellular proliferation, adhesion, and matrix remodeling. The study highlights the potential of waveform collagen microfibers as scaffolds for periodontal ligament tissue creation by highlighting the biomimetic environment they can create to support PDL cell differentiation and function [39].

Another unique application of 3D printing is the fabrication of interim and definitive maxillofacial obturators. Obturators are synthetic structures that are typically used to fill in the spaces created in the maxilla, mandible, and other skull bones following resection procedures indicated in the cases of cancer, trauma, or congenital defects. 3D-printed obturators were clinically investigated in a clinical study [40], where a 65-year-old patient had undergone bilateral maxillectomy to treat squamous cell carcinoma. The study’s method involved creating a stereolithographically 3D-printed plastic model of the defect using CT imaging and 3D planning software (SimPlant v14, Materialise Dental N.V., Leuven, Belgium), which served as a guide for building a hollow-bulb obturator. This model was used to create a wax pattern for the bulb, which was then lined with heat-cured silicone and used to take a final impression of the defect. This process resulted in a hollow space inside the obturator during processing and the duplication of the master cast. To enhance the bond, clear acrylic resin was sandwiched around the soft liner. Following the insertion of the obturator in the patient’s mouth, the prosthesis was relined chairside when necessary, and the patient experienced no problems during a five-year follow-up. The authors argue that a reliable and patient-friendly method of creating definitive obturators, or prosthetic fabrication, can be achieved by integrating digital imaging (CT + 3D printing) with standard laboratory techniques. This method is particularly helpful in situations where traditional impressions are challenging because of pain, fibrotic tissue presence, or limited mouth opening [40].

Similarly, a study by [41] introduced a clinical method that creates an interim hollow-bulb maxillary obturator for patients following maxillectomy using postoperative CT imaging and 3D printing. The clinicians utilized the patient’s CT image to digitally rebuild the defect and create a highly accurate 3D-printed anatomical cast instead of the traditional impression methods, which can be painful or unsafe in a fresh surgical site, given the presence of several bony undercuts where impression materials may be caught. However, in this particular case, the structure of the obturator was more complex, as it involved two pieces: a lightweight hollow bulb that fits into the defect, in addition to a separate palatal part that restores the oral surfaces. The method was found to improve anatomical accuracy, minimize patient discomfort, and provide a stable interim prosthesis throughout the challenging healing process [41].

A fully digital method for creating a maxillary obturator prosthesis using a 3D-printed polyetheretherketone (PEEK) framework was presented in another paper [42]. A Computer-Assisted Design (CAD) model of the obturator was created following a digital scan of the patient’s oral cavity. The PEEK-based structure was produced directly from the CAD design after an initial resin cast was 3D printed as a working model. The final obturator was processed on the definitive cast, which was produced using a secondary functional impression. In comparison to traditional metal frameworks, the resulting PEEK framework demonstrated a more accurate fit, superior retention, and reduced weight, thereby enhancing patient comfort without compromising functionality. This method demonstrates the potential of digital workflows and innovative polymers in terms of creating precise, personalized, and clinically useful craniofacial prostheses [42].

To date, no published studies to date have discussed the 3D bioprinting of maxillofacial obturators employing living, cell-laden structures.

To summarize, as shown in Table 2, researchers investigating 3D bioprinting/3D printing in dental tissue regeneration predominantly used DPSCs and PDLCs. Hydrogel-based and composite bioinks, often supplemented with growth factors or ECM components, combined with extrusion bioprinting processes, were most effective in promoting cell viability, proliferation, and differentiation. These techniques showed promising outcomes in fabricating craniofacial prostheses and the regeneration of alveolar bone, periodontal tissues, and dentin-pulp complexes, establishing both functional restoration and structural integrity. Dentin-derived hydrogel bioinks have provided a biologically similar environment for the bioprinting of cell-laden constructs [35], and printing parameter optimization has shown that it is feasible to create PDL-containing constructs with controlled structural properties [36]. The potential of spatially regulated cell deposition to support localized development of human DPSCs and replicate components that make up the structure of native dental tissues is further demonstrated by the bioprinting of dentin–pulp complexes [37]. Similarly, by facilitating cell distribution and providing structural guidance for periodontal ligament repair, bioactive composite scaffolds and collagen-based printed structures have shown promise for periodontal tissue regeneration [38,39].

Despite these promising results, the evidence is primarily preclinical, with the majority of studies focusing on scaffold performance, in vitro cell behavior, or differentiation rather than proving long-term functional regeneration in therapeutically relevant models. Direct comparability and reproducibility between studies are further complicated by variations in cell sources, bioink composition, printing parameters, scaffold architecture, and characterization techniques. Specifically, despite the localized cellular differentiation observed in the dentin-pulp bioprinting method [37], the ability to replicate the intricate structure, vascularization, innervation, and functional integration of a mature dentin-pulp complex remains challenging. Similarly, bioprinting applications in periodontal tissue necessitate not only cell viability and differentiation but also repair of the periodontal attachment apparatus and stable integration with the surrounding periodontal tissues [36,38,39].

In addition to tissue regeneration, prosthetic dentistry has seen a rise in the use of conventional 3D printing, particularly for the fabrication of patient-specific maxillofacial obturators. A further advanced clinical use of additive manufacturing can be observed in the studies around 3D-printed obturator prosthetics [40,41,42]. The results of these studies show how digital design and 3D printing may be used to create patient-specific definitive, interim, and PEEK-based maxillary obturator prostheses. However, since the printed devices are passive prosthetic replacements rather than cell-laden constructs intended to regenerate living tissue, these applications should be distinguished from bioprinting-based tissue regeneration. Therefore, their clinical application shows that 3D fabrication using digital workflows is feasible in dentistry, but stops short of offering proof of biological tissue regeneration.

### 3.3. Tracheal Regeneration

Airway blockage is frequently caused by conditions affecting the trachea, such as stenosis, tracheomalacia, traumatic injuries, and papillary thyroid carcinoma. Given the consequences of these conditions, the need for novel biomaterials to support tissue regeneration becomes evident. Recent advancements in 3D bioprinting have facilitated the process of developing customized, biocompatible, cell-laden constructs that can precisely encourage cell development and tissue repair [43]. In an early study by Valle-Pérez et al. [43], an ultrashort peptide-based bioink with self-healing and elastic properties for 3D bioprinting of trachea-like structures was developed. The optimized peptide mixtures were found to effectively support mesenchymal stromal cell differentiation into chondrocytes, enabling the formation of elastic cartilage-like tracheal tissue. Further, the developed bioinks demonstrated high printing accuracy, robust mechanical performance, and superior support for cell viability and chondrogenesis. When maintained in culture for up to 100 days, the 3D bioprinted trachea-like structure maintained its flexibility and mechanical stability. This work presents an innovative path for the development of advanced, self-healing biomaterials for tracheal tissue engineering and regeneration, as it is the first to combine SPM with 3D bioprinting to construct a tracheal model [43].

The concept of tissue-engineered trachea (TET) was introduced by Gao et al. in 2017, with the purpose of treating long-segmental tracheal abnormalities [44]. The model was constructed using a 3D-printed PCL scaffold [44] with a reticular structure that closely resembled the architecture of a rabbit’s natural trachea. The dimensions of the scaffold were as follows: 1.65 cm in length, 5 mm in luminal diameter, and 1.5 mm thick per convolution. To promote the production of cartilage tissue, the scaffold was subsequently seeded with chondrocytes and cultured for two to four weeks. After being implanted into mice and rabbits, the designed scaffold showed the characteristics of mature cartilage. The results of this study highlight the potential of 3D-printed PCL scaffolds in advancing tissue engineering applications and reconstruct tracheal systems [44].

In 2019, Gao et al. The author of [45] further enhanced their TET model by utilizing a 3D-printed poly (L-lactic acid) (PLLA) scaffold. Similar to their 2017 experiments, autologous auricular chondrocytes were also utilized to seed the PLLA scaffold, which was fabricated to closely resemble the morphology of a rabbit’s natural trachea. To promote tissue maturation and vascular integration, the chondrocyte-seeded scaffold was dynamically pre-cultured in vitro for two weeks before being pre-vascularized in vivo via pedicled muscle flaps for another two weeks. This method produced a segmental tracheal substitute that resembles the native trachea in terms of mechanical characteristics and structure. After being implanted into rabbits with segmental tracheal abnormalities, the 3D-printed tracheal substitute showed improved cellular viability and rapid epithelialization due to the stable blood supply that the pedicled muscle flaps provided.

Similarly, Xia et al.’s study [46] described the process of fabricating a TET by seeding autologous auricular cartilage cells onto a 3D-printed PCL scaffold using a different animal model than rabbits. Autologous auricular chondrocytes were used to seed the PCL scaffold, which was made to closely resemble the shape of a goat’s natural trachea. The goal was to develop bioprinted structures that address long-segmental tracheal abnormalities. Following Gao et al.’s protocol, the chondrocyte-seeded scaffold was dynamically pre-cultured in vitro for two weeks before being pre-vascularized in vivo utilizing pedicled muscle flaps for a further two weeks. The results were that the culturing process produced a segmental tracheal substitute that resembles the native trachea in terms of mechanical characteristics and structure. Overall, the results of these three studies provide evidence supporting the potential of combining 3D-printed scaffolds, in vitro pre-culture, and in vivo pre-vascularization [45] for clinical applications of restoring long-segmental tracheal lesions.

In addition to studies reporting the use of 3D bioprinted scaffolds, scaffold-free bioprinting is also considered unique since it depends entirely on the cells themselves to produce tissue, without utilizing any synthetic or natural supporting biomaterial. What makes it remarkable is that cells may self-assemble, interact, and build their own ECM in a way that closely resembles natural tissue formation, which often contributes to improved biological fidelity and fewer issues with foreign-material reactions. Thus, a group of researchers developed a unique scaffold-free method for using 3D bioprinting technology in the synthesis of artificial tracheas [47]. Spheroids, made of several cell types, such as chondrocytes, MSCs, and endothelial cells, were used as bioinks by the bio-3D printer to create three-dimensional structures. Layer by layer, these spheroids were assembled to create a trachea-like graft, which was subsequently grown in a bioreactor to promote tissue growth. With an average tensile strength of 526.3 ± 125.7 mN, the designed tracheal grafts showed adequate mechanical strength. Histological analysis also revealed chondrogenesis and vasculogenesis. Without the use of immunosuppressants or foreign materials, the scaffold-free grafts were effectively transplanted into F344 rats, resulting in functional tracheal repair. The potential of scaffold-free bioprinting in synthesizing autologous tissue-engineered tracheas for airway restoration is demonstrated by this study [47].

In summary, advancements in 3D bioprinting have enabled the fabrication of both scaffold-based and scaffold-free tracheal structures that closely replicate the native trachea in form and function. For the repair of long-segmental tracheal lesions, scaffold-based methods that use materials like PCL and PLLA seeded with chondrocytes or MSCs have shown promise in terms of cartilage creation, vascular integration, and mechanical stability. The additional advantage of scaffold-free approaches, which rely on cell spheroids that self-assemble and create their own ECM, is that they can accomplish chondrogenesis and functional airway regeneration without the risk of foreign material reactions. Collectively, these investigations, shown in Table 3, illustrate the vast promise of 3D bioprinting technology in developing customized and physiologically realistic tracheal alternatives, opening the path for future clinical applications in airway reconstruction.

These studies show complementary approaches to tracheal regeneration. While scaffold-based methods offer a predetermined three-dimensional framework that can support tissue growth and mechanical integrity [44,45,46], bioink-based methods offer direct spatial deposition of living cells within a printable biomaterial [43]. Given the promising in vitro results of tracheal regeneration using in vitro models, the transition to rabbit and goat models is expected to be relatively rapid. Furthermore, the scaffold-free bio-3D printing technique offers an alternative approach to construct tracheal tissue from cellular components alone. Overall, current available evidence proposes scaffold-based 3D printing as an approach to facilitate help in tracheal tissue development. However, the majority of comprehensive studies currently utilize preclinical animal research rather than clinical applications in humans.

### 3.4. Laryngeal Regeneration

The purpose of laryngeal regeneration is to restore the speech, breathing, and swallowing functions in patients who have had a laryngectomy due to cancer, trauma, or congenital defects. Tissue engineering and 3D bioprinting approaches that combine scaffolds, bioactive hydrogels, and stem or chondrocyte cells are becoming more prevalent given the challenges of donor site morbidity and restricted tissue availability [48] currently experienced in patients opting for traditional treatment modalities.

In 2022, a pilot study was conducted to investigate the viability of using 3D-bioprinted airway grafts for reconstructive surgery in an ovine (sheep) model [49]. Using computer-assisted modeling, the study describes the process of designing laryngeal and tracheal grafts and later using additive manufacturing, a fabrication technique for cell-free scaffolds or structures using materials such as polymers, ceramics, or composites, to produce scaffolds made entirely of PCL. Prior to implantation, these PCL scaffolds were cultured in vitro for up to 14 days using the animals’ own MSCs. These grafts were used to perform anterior laryngotracheal reconstruction (LTR) on five sheep. Follow-up endoscopic and histological examinations conducted over the course of several weeks indicated that the cell-seeded PCL grafts integrated well in the two sheep who completed the trial. Specifically, respiratory epithelium grew on the lumen, suggesting the potential for tissue regeneration [49].

Following the success of 3D bioprinting of laryngeal tissues demonstrated in the aforementioned study [49], bioprinting was introduced in the area of laryngeal regeneration. A study conducted in 2023 [48] reported the process of synthesizing a cell-filled artificial larynx using an extrusion-based bioprinter, where a combination of a PCL outer framework (with 200–500 μm pores for strength and nutrient diffusion) and an inner hydrogel bioink composed of GelMA 7 wt% and glycidyl-methacrylated hyaluronic acid (GMHA) 5 wt% containing rabbit chondrocytes was utilized. The study described the development of a new fluidics supply (FS) system that continuously provided baseline medium into the printing bath. By limiting hydrogel dehydration and lowering thermal stress from hot PCL, this method helped preserve high cell viability. According to rheological testing, the bioink was found to exhibit shear-thinning and photopolymerization under UV light, and mechanical testing validated its stability when combined with the PCL frame and upregulation of cartilage-specific genes (aggrecan, SOX-9, COL2) [48].

To further expand research around laryngeal bioprinting, bone marrow-derived MSCs from ferrets (fMSCs) were obtained, and their multipotent potential (i.e., chondrogenic and osteogenic) was confirmed. Then, using a mixture of GelMA bioink loaded with fMSCs and PCL filaments to offer mechanical strength, the study reports the development of multi-material 3D bioprinted scaffolds. Following the printing of each layer, the GelMA was photocrosslinked (405 nm), and PCL was printed at 85 °C. In vitro results demonstrated that these structures showed stable DNA content over 21 days, high cell viability (about 75–85% viable cells immediately after printing), and signs of chondrogenic differentiation (increased collagen II deposition and glycosaminoglycan [GAG] synthesis). The results of the study showed that bioprinted grafts can enhance airway healing, lumen mucosalization, and an average airway volume expansion of about 8% when implanted into ferrets in an anterior laryngotracheal reconstruction model. However, the explanted grafts contained very few mature chondrocytes, indicating the need for further optimization of the model [50].

In summary, 3D bioprinting/3D printing has demonstrated remarkable potential for laryngeal tissue regeneration by enabling the fabrication of constructs that resemble the complex structure and function of the natural larynx. Scaffold-based techniques, using biocompatible polymers seeded with mesenchymal stem cells, have shown successful chondrogenic and osteogenic formation. Together, these advancements, as shown in Table 4, highlight the potential of 3D bioprinting for developing functional laryngeal structures that are patient-specific, thereby setting a foundation for future research and clinical applications in airway and voice restoration.

Investigators created a fluidic-integrated 3D bioprinting method intended to preserve cell viability during the development of laryngeal constructs [48]. In a 2022 pilot study [49], the researchers examined 3D bioprinting in airway reconstructive surgery, offering a preliminary evaluation of its possible use in restorative treatments [49]. Further, investigators created cartilage grafts loaded with ferret MSCs using 3D bioprinting and assessed them in a ferret surgical model of laryngotracheal repair [50]. Collectively, these studies demonstrate progress in the evaluation from cell viability towards surgical evaluation of cell-laden constructs in an animal model. A crucial step in assessing the effectiveness of bioprinted structures in a physiologically simulated environment is the use of a conventional surgical repair model with cartilage grafts loaded with stem cells. However, despite the promising results, these studies are not without their limitations. These limitations include the predominance of preclinical study designs in the current available evidence, combined with the small experimental scale, and the absence of long-term data on graft integration, mechanical stability, airway function, and safety. These results underline the need for additional validation in larger animal models and future clinical trials, but they still support the possibility of 3D bioprinting for laryngeal and laryngotracheal repair.

### 3.5. Ocular Regeneration

More than 4.2 million individuals worldwide experience vision impairment related to corneal diseases, representing a pressing health concern that is further complicated by the lack of donor corneas, which limits surgical interventions [51]. The development of 3D printing or bioprinting provides a promising solution for corneal tissue engineering, providing precise control over dimensionality, structural organization, and cell–matrix interactions. The choice of biomaterials and printing techniques is critical to determine the properties of 3D-printed corneal constructs [1,52].

A recent study by Ulag et al. [52] explored the potential of 3D printing to create a customized artificial corneal stroma substitute using a poly (vinyl alcohol) (PVA) and chitosan (CS) combination. The PVA/CS material was deposited into a dome-shaped corneal architecture with a thickness of 0.4 mm and a curvature that resembled the human cornea using fused filament fabrication (FFF) or an equivalent 3D printing technique such as indirect molding. This technique of indirect molding involves the utilization of a template that is used to fabricate the final shape of the scaffold. In this study, an aluminum mold was used for indirect molding. The bioink was prepared using 13 wt% PVA and CS at different small addition levels (1, 3, and 5 weight percent). The bioink was then characterized using SEM for morphology, FTIR for chemical composition and interaction validation, UV-visible spectrophotometry for optical transmittance, swelling and degradation tests (up to about 30 days), and mechanical testing for tensile strength under intraocular pressure conditions. Human adipose-derived mesenchymal stem cells (hADSCs) were found to survive after 30 days of incubation on the constructs, suggesting potential for stromal-layer differentiation. The study’s findings reveal that adding CS can reduce light transmittance and tensile strength, but the constructs still maintained normal intraocular pressure (12–22 mmHg) and demonstrated high biocompatibility. Overall, this study shows the feasibility of rapid, custom-shaped synthetic corneal stroma scaffolds that combine transparency, mechanical support, and cytocompatibility [52].

Moreover, study [53] presented the design and construction of a dome-shaped 3D-printed convex corneal implant, with the purpose of enhancing corneal stromal regeneration and re-epithelialization. To ensure a smooth curvature and a step-free surface, the study employed a GelMA bioink and type I collagen (10% *w*/*v* GelMA with 0.6% *w*/*v* collagen) that was extruded via a pneumatic dual-extruder 3D printer under temperature-controlled conditions (nozzle at ~27.5 °C, build-plate at ~20 °C). To stabilize the hydrogel, the components were UV-crosslinked (200 mW cm^−2^, 45 s) after printing. The physical characteristics of the implants were comparable to those of the native cornea: ~83% transmittance at 750 nm, ~51 kPa tensile strength, ~130 kPa compressive modulus, and ~83% water content. Rabbit corneal epithelial cells (RCECs) were seeded across regions of varying slope gradient on the convex implant and on flat controls to investigate the effects of curvature on epithelial cell behavior. The study highlights that steeper slopes resulted in greater cell alignment, nuclear/chromatin deformation indicative of mechanosensing via the RhoA–LmnA7 pathway, increased focal adhesion formation (vinculin up-regulation), and higher adhesion force (~555 nN vs. ~36.5 nN on flat). The alignment and adhesion enhancement were explained by finite element modeling, which confirmed that a larger slope gradient increases in-plane shear stress and traction forces on the cell layer. The convex implants demonstrated faster epithelial closure, greater conformity to adjacent tissue, stronger adhesion (as demonstrated by ZO-1, CK3, and vinculin immunostaining), and more organized stromal collagen V deposition when compared to flat implants and defect-only controls in an in vivo rabbit lamellar keratectomy model. Further, the convex implants demonstrated lower α-SMA and, by extension, less scarring, and neural fiber regeneration by day 180. Therefore, the study identifies hydrogel bioink based on GelMA/collagen, the 3D printing technique, and a biomimetic convex geometry as factors that can improve stromal tissue regeneration and epithelial adherence [53].

Bioprinting of corneal structures was also expanded to include human corneal keratocytes, as described in Isaacson, Swioklo, and Connon’s study [54]. The study reported the use of a low-viscosity collagen-based bio-ink loaded with human corneal keratocytes and extrusion-based 3D bioprinting. A bath technique to create a prototype of corneal-stromal substitute was also used. Using the freeform reversible embedding of suspended hydrogels (FRESH), the researchers described a bioprinting process that aims to produce high-resolution structures, where collagen is printed directly into a supporting bath composed of densely packed gelatin microparticles. The bioink was made from locally prepared type I collagen and encapsulated keratocytes, and was used to print constructs that replicated the native stromal curvature and layer geometry using a digitized human corneal model. The extruded collagen was found to retain its shape during the heat gelation process, as this support material functions as a reversible scaffold. Stable and intricate collagen structures that are usually impossible to synthesize were found to remain following the careful melting process of gelatin particles after printing. The collagen was cross-linked following the printing process, and the support material was then removed. The encapsulated keratocytes exhibited significant cell viability (over 90% on day 1 and approximately 83% on day 7). The potential to generate an equivalent of the cell-rich corneal stroma was validated by morphological and viability evaluations [54].

Building on Isaacson, Swioklo, and Connon’s study [54], He et al. [55] developed a 3D-printed bilayer corneal implant that resembles the natural stroma and epithelium. The dome-shaped structure was created using a digital light processing (DLP) 3D printing method and a photocrosslinked bioink made of poly (ethylene glycol) diacrylate (PEGDA) and GelMA. Rabbit corneal epithelial cells comprised the upper epithelial layer, but rabbit adipose-derived mesenchymal stem cells were found in the stromal layer. The hydrogel structure’s high accuracy and transparency were made possible by DLP printing. In vitro experiments verified exceptional cell survival and epithelial regeneration. Similarly, in vivo transplantation in rabbit corneas demonstrated rapid epithelial healing, good integration, and minimal scarring. Overall, the study showed that a GelMA-PEGDA bilayer hydrogel implant created using DLP can effectively promote corneal regeneration and restore transparency [55].

In an effort to improve tissue remodeling and cell interaction using biomaterials added to GelMA that was utilized in previous studies, Wang et al. [56] developed a 3D-printed corneal scaffold made of GelMA modified with hyaluronic acid (HA). To enhance the scaffold’s biological characteristics, HA was added to the GelMA matrix to produce the bioink and was found to improve the properties of the primary corneal stromal cells (CSCs) inside the scaffolds. According to the study, HA’s modification of GelMA made it possible to 3D print corneal scaffolds and enhanced the properties of the primary corneal stromal cells inside the scaffolds [56].

In addition, Wang et al. [57] described the use of DLP 3D bioprinting techniques to create a biomimetic bilayer limbal implant in their 2025 study. Corneal epithelial cells (CECs) and corneal stromal stem cells (CSSCs) were injected into the bioink GelMA and PEGDA. The resulting hydrogel demonstrated strong mechanical properties, high transparency, and appropriate swelling and degradation rates for ocular applications. The encapsulated CECs and CSSCs remained viable and exhibited proliferative activity in vitro. In a model of limbal stem cell deficit (LSCD), the corneal surface was successfully restored by hydrogels loaded with CECs and CEC/CSSC. In contrast to the CEC-loaded group, the CEC/CSSC-loaded group notably showed reduced corneal opacity and neovascularization, as well as quicker epithelial repair. These results demonstrate that 3D bioprinting may be used to create the limbal structure, and they imply that CEC/CSSC-loaded limbal implants may be used as a treatment for corneal blindness and LSCD [57].

Collectively, bioprinting and 3D printing have become viable methods for corneal tissue engineering, helping to precisely restore the corneal architecture and addressing the global scarcity of donor corneas. Scaffold-based techniques using biomaterials such as PVA/chitosan, GelMA/collagen, PEGDA/GelMA, and GelMA/HA were used to enable high cell viability, proliferation, and differentiation of corneal stromal cells, epithelial cells, MSCs, and keratocytes. Techniques including extrusion-based printing, FRESH, and DLP printing allowed fabrication of dome-shaped, multilayer, and biomimetic structures with appropriate transparency, mechanical strength, and curvature for stromal and epithelial regeneration. These results reveal that bioink composition, printing technology, and implant geometry critically impact cell activity, tissue organization, and functional outcomes, highlighting the possibility of 3D bioprinting for customized corneal repair and restoration of vision as shown in Table 5.

Early research showed that 3D printing can be used to fabricate artificial corneal structures [52], while later methods used biomimetic geometry and tissue-specific structural elements to more closely mimic the natural cornea [53]. These methods have been improved by cell-laden bioprinting, which makes it possible to develop corneal stromal substitutes with living stromal cells [54]. Multiple corneal layers, including the stroma and epithelium, have been integrated into a single 3D-printed implant in more intricate structures [55]. Furthermore, corneal stromal cells’ capacity to remodel printed GelMA–HA scaffolds shows that cellular activity may contribute to the engineered matrix’s maturation [56].

Despite the promising results in the area of ocular regeneration, there is limited evidence on long-term biocompatibility, mechanical stability, transparency, vascularization, immunological responses, and functional integration following implantation. In fact, these investigations remain mostly restricted to in vitro and preclinical models. Prior to clinical translation, challenges with repeatable cell dispersion, vascular-free tissue maturation, manufacturing scalability, and regulatory standardization must also be addressed via additional validation in long-term and clinically relevant models.

### 3.6. Muscle Regeneration

Microfibril structures are present across various tissues in the human body, including tendons, muscles, and neurons in the head and neck region. The majority of micropatterned fabrication techniques, however, have focused on 2D surface-patterned topologies that mimic the fusion and alignment of skeletal and cardiac muscle cells. The creation of realistic 3D microfibril structures is described as a persistent challenge, despite the advancement of 2D techniques. A 2018 study [58] aimed to create a micropatterned PCL microfiber strut that mimics the alignment and fusion of skeletal muscle cells in vitro using microfibrillating and leaching PVA from a PVA/PCL combination. A number of variables, such as a mixture ratio, processing temperature, and pneumatic pressure, were taken into consideration in order to achieve the ideal processing conditions. Type-I collagen was added to the fabricated structure to improve the microfibrous PCL bundle’s biocompatibility. The cellular reactions of the fabricated structure were determined using myoblasts (C2C12 cells). The analysis of myogenic differentiation and cell proliferation confirmed that the hybrid microfibrillated structure may be a significant platform for achieving effective muscle cell regeneration [58].

Prior to the aforementioned study, Choi et al. [59] described the process of 3D cell printing using bioink based on skeletal muscle ECM and functioning skeletal muscle tissue. A multi-head 3D bioprinter was utilized to deposit C2C12 myoblasts, decellularized ECM bioink produced from skeletal muscle, and poly(ε-caprolactone) (PεCL) to provide mechanical support. Collagen and glycosaminoglycans, which are native ECM components, were found to be retained by the muscle-derived bioink, which encouraged cell attachment and development. The constructs were printed in a layered fashion, with the bioink creating a biomimetic milieu for myoblast growth, and PεCL acting as a framework to preserve shape fidelity and porosity. After printing, the tissues were cultured under myogenic differentiation conditions, which resulted in the development of contractile function upon electrical stimulation, aligned myotube formation, and enhanced expression of myosin heavy chains. This study showed that skeletal muscle ECM-based bioink, in conjunction with hybrid printing of PεCL and cells, could produce structurally stable, bioactive, and functional skeletal muscle structures [59].

In 2019, Capel et al. [60] developed a scalable and reproducible method that employs 3D-printed molds to construct human skeletal muscle tissue. Polylactic acid (PLA) was employed as the printing material in the fused deposition modeling (FDM) 3D printing process to create the molds, which made it possible to create reusable, affordable, and adaptable casting frameworks for muscle tissue constructs. In order to create aligned and contractile muscle fibers, the molds were utilized to shape a fibrin-based hydrogel seeded with human skeletal muscle precursor cells (hMPCs), as opposed to printing the tissue directly. Electrical and mechanical stimulation allowed the tissue-engineered constructs to grow, promoting cellular alignment, fusion, and the production of myogenic markers such as myosin heavy chain. The functional tests also confirmed that the method was physiologically sound, revealing that the modified muscles could produce contractile force when stimulated [60].

To further optimize the process of bioprinting specifically for muscular bioprinting, Distler et al. [61] created a bioink based on oxidized alginate-gelatin, or ADA-GEL, and printed scaffolds loaded with mouse myoblast cells (C2C12) using an extrusion-based technique. The study examined the impact of bioprinting parameters, particularly the nozzle size and extrusion pressure, on the shear stress that the cells endured during printing. Additionally, the study examined the role of these parameters in shaping the orientation, migration, and differentiation of the cells. The ADA-GEL hydrogel was selected due to its compatibility with myogenic differentiation, biodegradability, and printability. The bioink-embedded cells were printed in distinct filaments, with the C2C12 cells aligned along the printing direction due to shear stresses during extrusion. In regions with a higher cell density, the cells were observed to gradually move towards the hydrogel surface and differentiate into organized myotube segments. The study demonstrates that cellular alignment and subsequent differentiation in a 3D-printed scaffold may be guided by adjusting printing settings, such as shear stress via nozzle/pressure [61].

In conclusion, as summarized in Table 6, both scaffold-based and bioink-based methods have advanced the fabrication of 3D microfibrillar structures for skeletal muscle regeneration. Research has shown that aligned myoblasts or human muscle precursor cells can multiply, fuse, and differentiate into functional myotubes using PCL/collagen microfibers, skeletal muscle ECM-based bioinks, fibrin hydrogels in 3D-printed molds, and ADA-GEL hydrogels. Printing parameters, bioink composition, and scaffold architecture were shown to influence cell alignment, contractility, and myogenic marker expression. These findings highlight the importance of both material selection and bioprinter settings in producing structurally stable and physiologically functional muscle tissues.

Several approaches were proposed to provide consistent topographical cues and encourage myoblast proliferation, alignment, and myotube formation. Among these approaches is the development of a 3D-printed microfibrillar scaffold [58], and the creation of cell-laden structures that incorporated living cells into an extracellular matrix bioink produced from skeletal muscle [59]. Another direction that was explored was the development of human tissue-engineered skeletal muscle models from primary human cells using scalable 3D-printed molds [60]. These investigations show a development from traditional 3D-printed scaffolds and molds that offer structural guidance to cell-laden bioprinting techniques that actively regulate cellular organization and differentiation and incorporate biological components during production.

These methods are nevertheless limited by their primarily in vitro and short-term evaluation, relatively simple tissue topologies, restricted vascularization and innervation, and the use of cell lines or materials originating from animals in certain investigations. Furthermore, translation toward clinically relevant skeletal muscle regeneration is still limited by challenges with bioink printability, cell viability, construct maturation, mechanical fidelity, and scalability.

### 3.7. Neural Regeneration

The rising prevalence of peripheral nerve injuries that affect motor and sensory abilities has led to the development of contemporary alternatives to autologous nerve grafts, which are limited by donor-site morbidity and limited graft availability [62]. In particular, nerve guide conduits, an emerging alternative to nerve autografts and allografts, have been widely explored as an advanced tool for the treatment of peripheral nerve injury [63]. This is particularly relevant in the head and neck region, as the abundance of peripheral nerves and terminal branches of cranial nerves and their proximity to areas where dentists and maxillofacial surgeons operate render them vulnerable to iatrogenic injuries [64].

One of the early attempts to explore the potential of bioprinting nerve conduits included PCL-based 3D porous nerve guidance conduits that were printed via Electrohydrodynamic jet 3D printing (EHD-jetting) [65]. By modifying process variables, EHD-jetting offers customizability, reproducibility, and scalability, thereby allowing for fine control of scaffold characteristics, including fiber diameter, pore size, porosity, and alignment. Five scaffold designs were created to assess the impact of pore size on mechanical behavior. The pore sizes ranged from 125 to 550 μm, and the porosities ranged from 65 to 88%. Scaffolds with 125 ± 15 μm pores demonstrated optimal properties in in vitro degradation testing, such as porosity above 60%, mechanical strength comparable to natural nerves, and a degradation pace in sync with the natural nerve regeneration intervals. According to RT-PCR analysis, scaffolds with 125 ± 15 μm pores showed the strongest cell proliferation and overexpression of important neural genes (NF-H, GAP-43, and β3-tubulin), which was supported by neural differentiation experiments. Furthermore, neurite alignment along the printed fiber direction was confirmed by immunocytochemistry for NF200 and β3-tubulin, suggesting that the EHD-jet 3D printed PCL scaffolds exceed conventional electrospun PCL structures in promoting neural development and differentiation [65].

In 2020, ref. [66] developed a 3D bioprinted nerve conduit that further advanced the [65] study by incorporating live platelets to improve the regeneration of peripheral nerves. An extrusion-based 3D printing technique was employed to create conduits made of GelMA combined with PEGDA as the main bioink and platelet-rich plasma (PRP) to provide sustained release of neurotrophic and angiogenic factors like NGF, VEGF, and PDGF. Multilayered tubular conduits with interior microchannels that facilitated axonal alignment and were found to match those of natural nerves were created during the printing process. The printed conduits’ strength and flexibility were examined via mechanical tests to ensure that they were equivalent to those of natural peripheral nerves. In vivo experiments using a rat sciatic nerve defect model showed accelerated axonal regeneration, improved myelination, and improved functional recovery compared to non-platelet controls. Similarly, in vitro studies revealed that platelet incorporation significantly increased Schwann cell proliferation and neurite outgrowth. Overall, the study demonstrated the therapeutic potential of incorporating live bioactive components into printed structures by establishing a platelet-functionalized 3D-printed GelMA/PEGDA conduit as a bioactive and mechanically supportive platform for successful peripheral nerve healing [66].

The restoration of the peripheral nerves’ physiological function was examined in a 2023 study [67], where a multiscale 3D-printed nerve guidance conduit was created with the purpose of replicating the electrical and structural characteristics of natural peripheral nerves. In order to create a hierarchical structure that supported both mechanical flexibility and electrical conductivity, a multi-material extrusion-based 3D printing technique was utilized to create conduits made of a biodegradable polyurethane (PU) matrix combined with conductive polypyrrole (PPy) nanoparticles and aligned PCL microfibers. This arrangement enabled topographical guidance and electrical stimulation to occur simultaneously in regenerated axons. The stability of the conductive network inside the printed scaffolds was facilitated by optimizing the bioink technology for both biocompatibility and electroactivity. When examining in vitro results, an improvement in Schwann cell adhesion, alignment, and neurite extension was observed. Similarly, in vivo implantation in a rat sciatic nerve lesion model (10 mm gap) indicated greater nerve regeneration, myelination, and functional recovery in comparison to non-conductive controls. According to the study’s findings, the 3D-printed PU/PCL/PPy multiscale conduit effectively mimicked the electrophysiological and microstructural conditions of peripheral nerves, thereby providing a tissue-engineering pathway for functional nerve regeneration and repair [67].

Overall, as shown in Table 7, the development of bioactive, structurally accurate, and mechanically supporting scaffolds is made possible by 3D bioprinting of nerve guidance conduits. Studies have reported using Schwann cells, neural stem cells, and supporting MSCs with bioinks such as GelMA/PEGDA, PCL, and PU/PCL/PPy composites to promote neurite alignment, axonal development, and electrical conductivity. Printing methods include EHD-jetting and multi-material extrusion printing, enabling precise control of fiber orientation, pore size, and microchannel design, resulting in increased in vitro Schwann cell proliferation, neurite extension, and in vivo nerve regeneration. Collectively, the findings demonstrate the potential of 3D-printed nerve conduits as viable, functional platforms for peripheral nerve regeneration in the head and neck region.

To create nerve guiding conduits for peripheral nerve repair that offer a structured channel to facilitate nerve regeneration, several approaches were proposed, including electrohydrodynamic jet 3D printing and the addition of live platelets to 3D-bioprinted nerve conduits. More recently, researchers have reportedly developed a conductive multiscale nerve guidance conduit with hierarchical fibers that combines structural guiding and electrical stimulation [67]. Collectively, these studies highlight the evolution of printed conduits into multifunctional structures that incorporate electrical and biological signals. However, the evidence is still primarily preclinical, and cross-study comparisons should be approached with caution, given the large-scale variation in conduit materials, methods of development, injury models, and outcome measures. Furthermore, additional validation of long-term regeneration, functional recovery, manufacturing repeatability, and performance in clinically relevant peripheral nerve injuries would be necessary for translation to clinical applications.

## 4. Limitations of 3D Printing/3D Bioprinting

Despite their potential and promising results, 3D bioprinting technology is not without its limitations, particularly in the area of stem cell research and clinical translation [68]. While 3D-bioprinted structures do demonstrate high levels of precision and accuracy, concrete evidence derived from preclinical and clinical studies to ensure bioprinted structures’ compatibility and safety is yet to be established [69]. Additionally, the shortage of mass production of bioinks and 3D-bioprinted structures remains a challenge experienced by contemporary regenerative medicine, and often translates to high bioprinting costs [70]. Further, some 3D-printed scaffold materials are cytotoxic, and their formulation process may include high risks of toxicity and pathogenicity, thereby limiting the ability to translate their in vitro findings to clinical regenerative medicine [71]. The complex geometric shapes of composite materials also pose a major challenge that persists in bioprinting, coupled with the need to process numerous materials and post-optimize composite surface properties by employing various 3D printing technologies [72,73]. Lastly, with the rapid development of stem cell regenerative medicine treatments, the social ethics, legal rationality, and regulatory issues brought by printing organs and tissues also need to be taken into consideration [74].

## 5. Conclusions

In conclusion, bioprinting and 3D printing are revolutionary developments in tissue engineering that have moved the field from the fabrication of static and passive scaffolds to the production of dynamic and cell-laden constructs that closely resemble the structure and function of original tissue. Customized regenerative therapeutics and advanced disease models are made possible by these technologies, which combine living cells, biomaterials, and bioactive factors to provide precise control over tissue architecture and cell function. The goal of synthesizing functional and patient-specific tissues and organs in the craniofacial region and beyond will become feasible as bioinks and printing techniques continue to advance, expanding their therapeutic and research potential.

## Figures and Tables

**Table 1:** Summary of the selected 3D printing/bioprinting studies on bone and cartilage regeneration, their clinical evidence, and the principal outcomes of the study.

3D-Printed/Bioprinted Construct	Clinical Evidence	Outcomes	Reference
3D-bioprinted bioink containing periodontal ligament stem cells (PDLSCs) and myeloid-derived suppressor cell membrane vesicles for periodontitis-related bone defects.	Preclinical (in vivo)	The multifunctional bioink provided antibacterial, anti-inflammatory, and osteogenic effects and promoted periodontal bone repair. The approach shows potential, but translation to humans remains unvalidated.	[25]
Triple-integrated 3D-printed composite scaffold containing α-TCP bone cement, ZnO nanoparticles, and peptide-loaded gelatin microspheres for diabetic bone defects.	Preclinical	The integrated scaffold was designed to address the difficult hyperglycemic/oxidative environment and promote combined osteogenic and vascular regenerative repair.	[26]
3D-printed ICIE16 bioactive-glass scaffolds seeded with human bone-marrow-derived stromal cells (hBMSCs).	In vitro	The 3D-printed bioactive-glass scaffold promoted osteogenic differentiation, with scaffold contact and ionic dissolution both contributing to the response. It supports the biological potential of the material.	[27]
Borosilicate bioactive-glass material subjected to crystallization and sintering for tissue-engineering applications.	Material/preclinical level	The study provides important information regarding the processing and crystallization behavior of bioactive glasses, supporting material optimization.	[28]
3D-printed resorbable bone implants intended for in vitro studies and in vivo bone repair.	Preclinical	The bone implant is a promise for customized bone regeneration. They are unique for their printability, biodegradability, and structural control.	[29]
3D-bioprinted cell-laden nanocellulose–alginate constructs containing human chondrocytes; anatomically shaped cartilage structures including an ear and sheep meniscus were printed.	In vitro	The nanocellulose–alginate bioink provided suitable printability and supported short-term viability of human chondrocytes, demonstrating potential for cartilage bioprinting.	[30]
3D-printed polymer scaffolds with controlled micro-/nanoporous surface topography for MSC culture.	In vitro	Printed micro-/nanotopography enhanced osteogenic and chondrogenic differentiation without soluble differentiation factors, demonstrating that scaffold architecture itself can influence cell fate.	[31]
Multiphasic osteochondral constructs incorporating nano-to-micro features using PCL-based bioink, combining 3D printing with casting.	Preclinical (in vitro)	The approach demonstrates the feasibility of reproducing the complex, graded architecture of osteochondral tissue.	[32]
3D-bioprinted synovium-derived MSC-laden photocrosslinked ECM bioink containing GelMA, HAMA, and chondroitin sulfate methacrylate for cartilage regeneration.	Preclinical	This study concludes that region-specific osteogenic and chondrogenic regeneration can be achieved via customized bioink compositions coupled with an integrated casting process to efficiently regulate the mechanical properties and cellular responses of osteochondral structures.	[33]
3D printed platform for articular cartilage regeneration.	Preclinical (in vitro)	The platform provides a 3D model for studying cartilage regeneration.	[34]

**Table 2:** Summary of the selected 3D printing/bioprinting studies in dental regeneration, their clinical evidence, and the principal outcomes of the study.

3D-Printed/Bioprinted Construct	Clinical Evidence	Outcomes	Reference
Dentin-derived hydrogel bioink used for 3D bioprinting of cell-laden scaffolds for regenerative dentistry.	In vitro	The dentin-derived hydrogel bioink supported cell viability and provided a biologically relevant microenvironment for 3D bioprinting, demonstrating potential for dental tissue regeneration.	[35]
3D-bioprinted PDL constructs, with optimization of printing parameters for PDL cell deposition.	In vitro	Optimized printing conditions enabled the fabrication of periodontal ligament cell-containing constructs while maintaining cell viability and supporting the development of organized 3D structures.	[3]
3D-bioprinted dentin–pulp complex using hDPSCs with spatially controlled differentiation.	In vitro	The bioprinted construct enabled localized differentiation of hDPSCs and demonstrated the feasibility of generating organized dentin–pulp-like tissue structures.	[37]
3D-bioprinted bioactive composite scaffold designed for cell delivery in periodontal tissue regeneration.	Preclinical (in vivo)	The bioactive composite scaffold provided a suitable platform for cell delivery and promoted periodontal tissue regeneration, supporting its potential as a regenerative periodontal construct.	[38]
3D-printed collagen-based waveform microfibrous scaffold designed to reproduce structural features of the PDL.	In vitro	The waveform microfibrous architecture provided structural guidance for periodontal ligament-related cellular behavior and demonstrated potential for PDL reconstruction.	[39]
3D-printed definitive maxillary obturator prosthesis fabricated using digital design and additive manufacturing.	Clinical case report	The study demonstrated the clinical feasibility of fabricating a definitive obturator prosthesis using 3D printing and digital workflows.	[40]
3D-printed interim obturator prosthesis fabricated through a digital workflow.	Clinical case report	The study demonstrated that 3D printing could be used to fabricate an interim obturator prosthesis, providing a contemporary digital approach to prosthetic rehabilitation.	[41]
3D-printed PEEK framework for a maxillary obturator prosthesis.	Clinical case report	The study demonstrated the feasibility of digitally fabricating a maxillary obturator prosthesis using a 3D-printed PEEK framework, supporting the clinical application of additive manufacturing in prosthodontic rehabilitation.	[42]

**Table 3:** Summary of the selected 3D printing/bioprinting studies in tracheal regeneration, their clinical evidence, and the principal outcomes of the study.

3D-Printed/Bioprinted Construct	Clinical Evidence	Outcomes	Reference
3D-bioprinted trachea-like constructs using mixed ultrashort peptide bioinks containing mesenchymal stromal cells.	In vitro	The optimized bioinks demonstrated good printability, self-healing, and elastic properties, while supporting cell viability and chondrogenic differentiation.	[43]
Tissue-engineered trachea based on a 3D-printed scaffold for whole-segment tracheal repair.	Preclinical (in vivo)	The 3D-printed scaffold-based tissue-engineered trachea enhanced repair of whole-segment tracheal defects and supported regeneration of tracheal tissue.	[44]
Pedicled tissue-engineered trachea based on a 3D-printed scaffold for long-segment reconstruction in rabbits.	Preclinical (in vivo)	The 3D-printed scaffold-based tissue-engineered trachea enabled long-segment tracheal reconstruction and supported tissue regeneration in rabbits.	[45]
Tissue-engineered trachea fabricated using a 3D-printed scaffold for whole-segment repair.	Preclinical (in vivo)	The study demonstrated the feasibility of using a 3D-printed scaffold-based tissue-engineered trachea for whole-segment tracheal repair in a large-animal model.	[46]
Scaffold-free bio-3D-printed tracheal tissue generated from cellular aggregates.	Preclinical (in vivo)	Scaffold-free bio-3D printing enabled the generation of tracheal tissue and demonstrated the potential of cellular self-assembly for tracheal regeneration.	[47]

**Table 4:** Summary of the selected 3D printing/bioprinting studies in laryngeal regeneration, their clinical evidence, and the principal outcomes of the study.

3D-Printed/Bioprinted Construct	Clinical Evidence	Outcomes	Reference
Fluidic-integrated 3D bioprinting system developed for fabrication of rabbit chondrocyte-containing laryngeal constructs while maintaining cell viability.	In vitro/proof-of-concept	The fluidic-integrated system successfully supported the fabrication of 3D bioprinted constructs while maintaining cell viability, demonstrating its potential for laryngeal tissue fabrication.	[48]
3D bioprinting approach for airway reconstruction, evaluated as a pilot study.	Pilot/preclinical translational study	The study demonstrated the feasibility of applying 3D bioprinting concepts to airway reconstructive surgery and provided preliminary evidence supporting further development of the approach.	[49]
fMSCs-laden 3D-bioprinted cartilage grafts for laryngotracheal reconstruction.	Preclinical, surgical animal model (ferret)	The study demonstrated the feasibility of using stem cell-laden bioprinted cartilage grafts for laryngotracheal reconstruction and provided evidence of their performance in a surgical model.	[50]

**Table 5:** Summary of the selected 3D printing/bioprinting studies in ocular regeneration, their clinical evidence, and the principal outcomes of the study.

3D-Printed/Bioprinted Construct	Clinical Evidence	Outcomes	References
3D-printed artificial cornea designed for corneal stromal transplantation.	Preclinical (in vitro)	The 3D-printed artificial cornea demonstrated suitable structural and material properties for corneal stromal transplantation, supporting the feasibility of 3D printing for artificial corneal development.	[52]
3D-printed biomimetic convex corneal implant designed to reproduce the geometry and function of the native cornea.	Preclinical (in vivo)	The biomimetic implant promoted corneal regeneration and demonstrated the potential of 3D printing to produce anatomically appropriate implants for corneal repair.	[53]
3D-bioprinted corneal stroma equivalent using a keratocyte-containing bioink to reproduce corneal stromal tissue.	In vitro	The study demonstrated the feasibility of 3D bioprinting a corneal stroma equivalent while maintaining corneal stromal cell viability and supporting the formation of a tissue-like stromal construct.	[54]
3D-printed biomimetic epithelium/stroma bilayer hydrogel implant for corneal regeneration.	Preclinical (in vivo)	The bilayer hydrogel implant reproduced key structural features of the cornea and promoted regeneration of both epithelial and stromal tissues, demonstrating potential for corneal repair.	[55]
3D-printed GelMA–HA corneal scaffold remodeled by corneal stromal cells.	In vitro	Corneal stromal cells were able to remodel the 3D-printed GelMA–HA scaffold, demonstrating cellular interaction with and modification of the printed corneal matrix.	[56]
3D-bioprinted biomimetic bilayer limbal implant fabricated by DLP using GelMA/PEGDA bioinks containing corneal epithelial cells and corneal stromal stem cells.	Preclinical (in vivo)	The cell-laden bilayer implant supported corneal epithelial regeneration in a rabbit model of limbal stem cell deficiency, with the CEC/CSSC-loaded construct producing faster epithelial healing and reduced corneal opacity and neovascularization.	[57]

**Table 6:** Summary of the selected 3D printing/bioprinting studies in muscle regeneration, their clinical evidence, and the principal outcomes of the study.

3D-Printed/Bioprinted Construct	Clinical Evidence	Outcomes	Reference
3D-printed biomimetic PCL microfibril scaffold designed to reproduce the aligned microfibrillar structure of skeletal muscle.	In vitro	The microfibrillar scaffold promoted myoblast proliferation, alignment, and myotube formation, demonstrating that biomimetic 3D architecture can provide structural cues for skeletal muscle tissue engineering.	[58]
3D-bioprinted skeletal muscle constructs using a skeletal muscle-derived decellularized ECM bioink containing cells.	In vitro	The muscle-derived ECM bioink supported high cell viability, cellular alignment, myotube formation, differentiation, maturation, and contractility, demonstrating the feasibility of producing functional skeletal muscle constructs by 3D cell bioprinting.	[59]
3D-printed molds used to fabricate human tissue-engineered skeletal muscle constructs from primary human skeletal muscle cells.	In vitro	The scalable 3D-printed molds enabled fabrication of tissue-engineered skeletal muscle models using primary human tissue, providing a reproducible platform for studying human skeletal muscle physiology and pathology.	[60]
3D-bioprinted C2C12 muscle precursor cell-laden oxidized ADA-GEL bioink	In vitro	Appropriate nozzle size and extrusion pressure generated shear stress that promoted C2C12 cell orientation along the printing direction, followed by myotube formation and differentiation. The ADA-GEL bioink supported in vitro skeletal muscle tissue engineering.	[61]

**Table 7:** Summary of the selected 3D printing/bioprinting studies on neural regeneration, their clinical evidence, and the principal outcomes of the study.

3D-Printed/Bioprinted Construct	Clinical Evidence	Outcomes	Reference
Electrohydrodynamic jet 3D-printed nerve guidance conduits (NGCs) for peripheral nerve repair.	Preclinical (in vivo)	The 3D-printed NGCs provided a suitable guidance structure for peripheral nerve regeneration and demonstrated improved functional nerve repair compared with the injury control.	[65]
3D-bioprinted nerve conduits incorporating live platelets for peripheral nerve repair.	Preclinical (in vivo)	The incorporation of live platelets provided a biologically active microenvironment that enhanced nerve regeneration and functional recovery, demonstrating the potential of biologically functionalized 3D-printed conduits.	[66]
3D-printed conductive multiscale nerve guidance conduit incorporating hierarchical fibers for peripheral nerve regeneration.	Preclinical (in vivo)	The conductive, hierarchical conduit promoted axonal regeneration, Schwann-cell activity, remyelination, and functional recovery, demonstrating the potential of combining electrical conductivity with multiscale structural guidance.	[67]

## Data Availability

No new data were created or analyzed in this study. Data sharing is not applicable to this article.

## Abbreviations

The following abbreviations are used in this manuscript:

Abbreviation	Full term
3D	Three-dimensional
ADA-GEL	Oxidized alginate–gelatin
ALP	Alkaline phosphatase
BGM	Bioactive glass microspheres
BMP-2	Bone morphogenetic protein 2
CAD	Computer-aided design
CAD/CAM	Computer-aided design/computer-aided manufacturing
CaCl_2_	Calcium chloride
CCK-8	Cell Counting Kit-8
CEC	Corneal epithelial cell
COL2	Collagen type II
CS	Chitosan
CSC	Corneal stromal cell
CSSC	Corneal stromal stem cell
CT	Computed tomography
DLP	Digital light processing
DMP-1	Dentin matrix protein 1
DNA	Deoxyribonucleic acid
DPSC	Dental pulp stem cell
DSPP	Dentin sialophosphoprotein
ECM	Extracellular matrix
EHD	Electrohydrodynamic
EHD-jetting	Electrohydrodynamic jet printing
FDM	Fused deposition modeling
FFF	Fused filament fabrication
FRESH	Freeform reversible embedding of suspended hydrogels
FS	Fluidics supply
GAG	Glycosaminoglycan
GAP-43	Growth-associated protein 43
GAS	Gelatin–alginate–silk fibroin
GelMA	Gelatin methacryloyl
GMHA	Glycidyl-methacrylated hyaluronic acid
HA	Hyaluronic acid
H&E	Hematoxylin and eosin
hADSC	Human adipose-derived mesenchymal stem cell
hBMSC	Human bone marrow stem cell
hDPSC	Human dental pulp stem cell
hMPC	Human skeletal muscle precursor cell
hMSC	Human mesenchymal stem cell
hNDF	Human neonatal dermal fibroblast
H_2_O_2_	Hydrogen peroxide
hPDLSC	Human periodontal ligament stem cell
HRP	Horseradish peroxidase
HUVEC	Human umbilical vein endothelial cell
LAP	Lithium phenyl-2,4,6-trimethylbenzoylphosphinate
LSCD	Limbal stem cell deficiency
LTR	Laryngotracheal reconstruction
mBMSC	Mouse bone marrow mesenchymal stem cell
MSC	Mesenchymal stem cell
MTT	3-(4,5-Dimethylthiazol-2-yl)-2,5-diphenyltetrazolium bromide
NFC	Nanofibrillated cellulose
NF-H	Neurofilament heavy chain
NGF	Nerve growth factor
nHA	Nanocrystalline hydroxyapatite
OCN	Osteocalcin
OPN	Osteopontin
PCL	Polycaprolactone
PDGF	Platelet-derived growth factor
PDL	Periodontal ligament
PDLSC	Periodontal ligament stem cell
PEG	Polyethylene glycol
PEGDA	Poly(ethylene glycol) diacrylate
PEEK	Polyetheretherketone
PLA	Polylactic acid
PLLA	Poly(L-lactic acid)
PεCL	Poly(ε-caprolactone)
PPy	Polypyrrole
PRP	Platelet-rich plasma
PU	Polyurethane
PVA	Poly(vinyl alcohol)
qRT-PCR	Quantitative reverse transcription polymerase chain reaction
RAD16-I	Self-assembling peptide RAD16-I
RCEC	Rabbit corneal epithelial cell
RNA-seq	RNA sequencing
RT-PCR	Reverse transcription polymerase chain reaction
Runx2	Runt-related transcription factor 2
SA	Sodium alginate
SEM	Scanning electron microscopy
SMSCs	Synovium-derived mesenchymal stem cells
SOX-9	SRY-box transcription factor 9
SPM	Self-assembling peptide material
TET	Tissue-engineered trachea
TGF-β1	Transforming growth factor beta 1
UV	Ultraviolet
VEGF	Vascular endothelial growth factor
α-SMA	Alpha-smooth muscle actin
α-TCP	Alpha-tricalcium phosphate
β-TCP	Beta-tricalcium phosphate
